# CT引导下Hook-wire精确定位并微创切除肺结节

**DOI:** 10.3779/j.issn.1009-3419.2015.11.04

**Published:** 2015-11-20

**Authors:** 通 王, 少华 马, 天生 闫, 金涛 宋, 可毅 王, 未 贺, 洁 白

**Affiliations:** 100191 北京，北京大学第三医院胸外科 Department of Thoracic Surgery, Peking University Third Hospital, Beijing 100191, China

**Keywords:** 肺磨玻璃样结节, 电视胸腔镜手术, CT引导下Hook-wire定位, Ground glass opacity, Video-assisted thoracic surgery, CT-guided Hook-wire localization

## Abstract

**背景与目的:**

肺小结节尤其是磨玻璃结节(ground glass opacity, GGO)病灶的定位是微创手术切除的难点，报道的方法很多但均有不足。本研究旨在探讨计算机断层扫描(computed tomography, CT)引导下Hook-wire术前定位在胸腔镜下(video-assisted thoracoscopic surgery, VATS)肺结节切除术中的临床应用价值，并初步探讨GGOs积极微创手术治疗的必要性和可行性。

**方法:**

2013年5月-2015年6月共25例患者的26枚肺结节于术前行CT引导下Hook-wire定位，然后施行胸腔镜楔形切除术。统计Hook-wire定位时间、成功率、并发症及楔形切除时间、住院时间等，计算病灶组织学分型中的恶性几率，讨论肺部GGOs积极手术治疗的必要性。

**结果:**

共25例患者26个结节(男性10例，女性15例，6个实性结节，20个GGOs)，病灶直径5 mm-20 mm(平均8 mm)，病灶距离胸膜垂直距离5 mm-30 mm(平均14 mm)，CT引导下Hook-wire定位成功率为100%。VATS楔形切除术成功率为100%。CT定位时间平均10 min(5 min-15 min)，微创切除病灶所需时间平均20 min(15 min-40 min)，平均住院时间为4 d(3 d-6 d)。4例患者定位后发生微量气胸，但无需闭式引流处理。术中定位针脱落1例，但仍于胸腔镜下观察到穿刺点脏层胸膜下血肿后，准确定位并成功切除。20个GGOs术后组织学诊断结果为：16个混合性GGOs(mixed GGO, mGGO)中，微浸润腺癌2例，腺癌5例，小细胞肺癌(small cell lung cancer, SCLC)1例，炎性病灶8例；4个纯GGOs(pure GGO, pGGO)中原位腺癌1例，非典型性腺瘤样增生(atypical adenomatoid hyperplasia, AAH)1例，炎性病灶2例。

**结论:**

CT引导下Hook-wire肺结节尤其是GGOs术前定位准确率高，相关并发症轻微，是一种安全、有效的方法，能快速确定下一步诊疗方案，值得临床推广；肺部mGGOs是恶性病灶的几率很大，积极微创手术治疗是非常必要的。

## Abstract (en)

**Background and objective:**

Localization of pulmonary ground glass nodule is the technical difficulty of minimally invasive operation resection. The aim of this study is to evaluate the value of computed tomography (CT)-guided Hook-wire precise localization in resection of pulmonary nodules by video-assisted thoracoscopic surgery (VATS) as well as to discuss the necessity and feasibility of surgical resection of GGOs through a minimally invasive approach.

**Methods:**

CT-guided Hook-wire precise localization and wedge resection was done on 25 cases with 26 pulmonary nodules by VATS from May 2013 to June 2015. The efficacy of localization was evaluated in terms of procedure time, VATS success rate, and associated complications of localization.

**Results:**

All the 26 pulmonary nodules (6 solid nodules and 20 GGOs) of 25 patients (10 males and 15 females) were preoperatively detected and localized with Hook-wire under CT guidance. Nodule diameters ranged from 5 mm-20 mm (mean: 8 mm). The distance of lung lesions from the nearest pleural surfaces ranged within 5 mm-30 mm (mean: 14 mm). All resections of lesions guided by the Hook-wire were successfully performed by VATS (success rate: 100%). The mean procedure time for the CT-guided Hook-wire localization was 10 min (range: 5 min-10 min). The mean procedure time for VATS wedge resection was 20 min (range: 15 min-40 min). The mean hospital time was 4 d (range: 3 d-6 d). The major complication of CT-guided Hook-wire localization was mild pneumothorax in 4 patients, but no one needed chest tube drainage. Wedge resection was performed successfully in all cases. The dislocation of Hook-wire was found in only one patient during the operation, but the lesion was still successfully resected under VATS. Results of pathological examination of 16 mGGOs revealed 8 primary lung cancers and 8 nonspecific chronic inflammations. Results of pathological examination of 4 pGGOs revealed 1 primary lung cancers, 1 atypical adenomatous hyperplasia (AAH), and 2 nonspecific chronic inflammations.

**Conclusion:**

The preoperative CT-guided Hook-wire localization for pulmonary nodules particularly for GGOs is an effective and safe technique to assist VATS resection of the nodules. It can increase the ratio of lung wedge resection with little complications and may be better used in clinical diagnosis and treatment of small pulmonary nodules with VATS. Lung mGGOs carry a high risk of malignancy. Aggressive surgical resection of these mGGOs is necessary and feasible.

随着多排螺旋计算机断层扫描(computed tomography, CT)的应用，临床上越来越多的孤立性肺结节(solitary pulmonary nodule, SPN)被检出。据2007年美国胸科医师学会(American College of Chest Physicians, ACCP)临床实践指南报道^[1]^：SPNs的检出率为8%-51%，1.1%-12%的SPNs为恶性，其中磨玻璃密度影(ground-glass opacity, GGO)高达59%-73%为恶性，多数为原位腺癌，亦可以是微浸润腺癌甚至是浸润腺癌，应当予以积极处理。电视胸腔镜(video-assisted thoracic surgery, VATS)肺楔形切除术可以微创、安全且完整地切除病灶，诊断准确率近100%，现已广泛应用于SPNs的诊断和治疗当中。由于SPNs尤其是GGOs有难以用手触知、不能肉眼发现的特点，胸腔镜下有时候很难准确定位，影响VATS的成功率并导致较高的中转开胸率^[2]^。如何术前对病灶进行精确定位是急需解决的难题。目前国内研究及报道较多的是CT引导下Hook-wire定位^[2-4]^。2013年5月-2015年6月我们对25例患者的26枚肺结节(其中20个GGOs)术前采用CT引导下Hook-wire定位的方法，取得较好的临床效果，现将结果报告如下。

## 1 资料与方法

### 1.1 临床资料

2013年5月-2015年6月，北京大学第三医院25例患者的26枚肺结节经术前CT引导下Hook-wire(美国Angiotech公司，Accura BLN乳腺定位针)定位后行VATS肺楔形切除术。男性10例，女性15例，年龄40岁-77岁，平均60岁，实性结节6个，GGOs 20个，其中混合性GGOs(mixed GGO, mGGO)16个，纯GGOs(pure GGOs, pGGO)4个。所有患者术前均进行恶性可能性筛查，入组标准为：影像学提示恶性程度高(如分叶、毛刺、血管集束征等)；或者观察期间结节增大或者实性成分增多；或者经抗炎治疗后无明显变化；或者有肺癌危险因素，如高龄、肺癌家族史或者其他恶性肿瘤病史、吸烟史等。其中1例女性有直肠癌病史，其余患者均体检发现，无恶性肿瘤病史。胸部薄层CT扫描提示1例患者为多发结节，其余均为单发结节，右肺13个，左肺13个。病灶直径平均8 mm(5 mm-20 mm)，病灶距离胸膜垂直距离平均14 mm(5 mm-30 mm)。

### 1.2 技术方法

所有患者先行胸部CT扫描，选择合适的体位、穿刺部位，确定进针的深度以及最佳的进针角度和路径。定位前肌肉注射哌替啶50 mg，常规消毒、铺无菌单，穿刺点以1%利多卡因局麻后穿入Hook-wire定位系统套针，重复CT扫描，显示套针位于病灶内，回抽无血，继续推进套针约5 mm，释放Hook-wire并回收套针，再次进行CT扫描，显示定位钢丝穿过结节，顶端超过病灶约5 mm。紧贴胸壁穿刺点剪断钢丝(最初我们曾距离胸壁穿刺点5 cm剪断钢丝，由于留余太长不利于转运及护理)，以无菌纱布覆盖，送患者入手术室。全麻后，健侧卧位，患侧朝上，常规消毒铺无菌单。做切口并置入胸腔镜(上叶病变经腋前线第6肋间进镜，腋前线第3肋间做操作孔；下叶病变经腋前线第7肋间进镜，腋前线第4肋间做操作孔；如需切肺叶则在腋后线第9肋间加做辅助孔)，全面检查胸腔，并伸入食指探查，结合影像确定钢丝深度和病灶部位。VATS手术时，结合影像并根据Hook-wire判断结节的具体位置，用抓钳提起定位钢丝，先以长轴卵圆钳试夹含钢丝及结节的肺组织，距钢丝尖端2 cm再以腔镜直线型切割缝合器楔形切除病灶，取出标本，送快速冰冻切片检查，根据病理结果决定下一步手术方案。如为原发性非小细胞肺癌(non-small cell lung cancer, NSCLC)且患者能耐受手术，则继续行VATS肺叶切除术加系统淋巴结清扫；若患者全身情况差，不能耐受肺叶切除术，则仅行肺楔形切除术。

## 2 结果

本组25例患者的26枚结节，CT引导下Hook-wire定位成功率为100%，VATS肺楔形切除术成功率为100%(图 1)。CT定位时间平均10 min(5 min-15 min)，均穿透病灶，定位满意。本组有4例穿刺后并发微量气胸，但无需闭式引流处理。全部患者术中均未发生血胸或肋间血肿。VATS楔形切除术成功率为100%，无中转开胸，无定位针残留，全部标本切缘距病灶均＞20 mm。术中定位针脱落1例，但仍于胸腔镜下观察到穿刺点脏层胸膜下血肿后，准确定位并成功切除。微创楔形切除病灶所需时间平均20 min(15 min-40 min)，平均住院时间为4 d(3 d-6 d)。本组11例患者术中冰冻病理诊断为原发性NSCLC，4例均成功施行VATS肺叶切除加淋巴结清扫术，其余患者因全身情况差、合并症多，仅行楔形切除术。20个GGOs术后组织学诊断结果见表 1：16个mGGOs中，微浸润腺癌2例，腺癌5例，小细胞肺癌(small cell lung cancer, SCLC)1例，炎性病灶8例；4个pGGOs中原位腺癌1例，非典型性腺瘤样增生(atypical adenomatoid hyperplasia, AAH)1例，炎性病灶2例。GGOs总的恶性比例是45%(9/20)，mGGOs为50%(8/16)，pGGOs则为25%(1/4)。6个实性结节术后组织学诊断结果为：炎性假瘤1例，鳞癌1例，浸润型腺癌1例，中分化腺癌1例，错构瘤1例，直肠癌转移1例。

**1:**
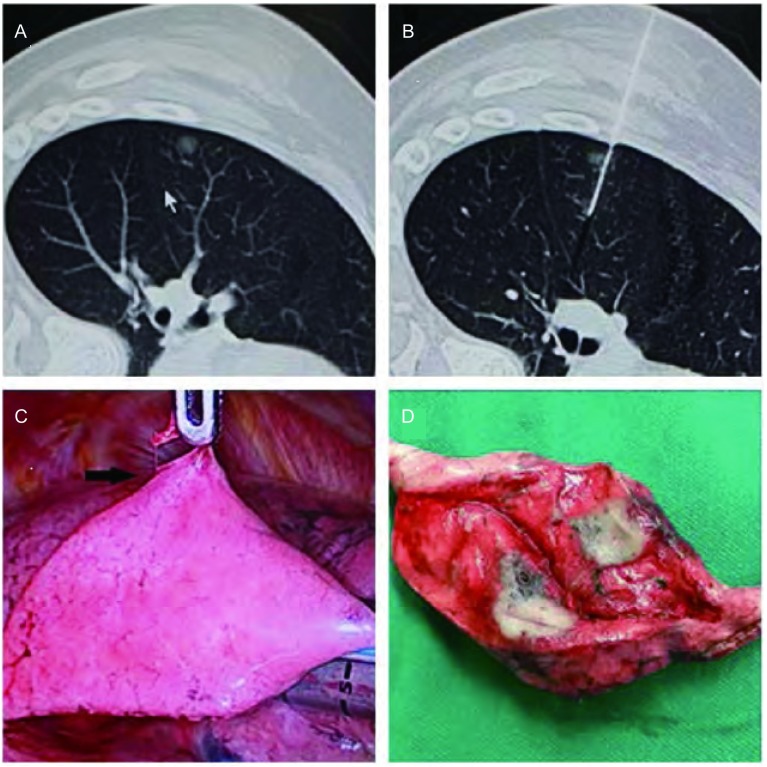
GGO病灶的CT定位扫描图及VATS切除。A：肺GGO病灶的CT扫描图；B：穿刺针定位于肺GGO病灶的CT扫描图；C：Hook-wire定位针引导肺GGO病灶的VATS切除；D：切除的肺组织中见直径7 mm病灶。 CT scanograms and VATS resection of the GGO lesionswith pilot pin. A: CT scanograms of the GGO lesions; B: CT scanograms of the GGO lesions with pilot pin; C: VATS resection of GGO lesions guided by Hook-wire pilot pin; D: Lesions (d=7 mm). VATS: video-assisted thoracic surgery; CT: computed tomography.

**1:** GGOs病理类型分析 Pathological analysis of GGOs

Pathological analysis	GGO (*n*)	Percent (%)
Primary lung cancer	9	45
AAH	1	5
Non-specific chronic inflammatory lesions	10	50
AAH: atypical adenomatoid hyperplasia; GGO: ground-glass opacity.		

## 3 讨论

VATS微创技术为SPNs尤其是GGOs的诊断提供了一个微创、快速、有效的方法，而影响VATS成功率的一个重要因素是能否快速、准确地找到病灶。病灶距胸膜表面的距离和病灶的直径决定能否快速、准确地被发现。病灶越小、距离脏层胸膜越远，术中的准确定位率越低。在没有术前定位措施的情况下，通常只能根据术前的CT判断病灶的大致解剖位置，伸入单个手指以及应用胸腔镜器械进行触查。虽然大部分病变能被找到并成功切除，但由于单肺通气、肺萎陷后解剖位置的改变以及某些病灶在密度、硬度上与周边肺组织难以区分等因素，仍有部分病灶不能找到^[3]^，尤其是距离胸膜比较远、亚厘米的SPNs，尤其是GGOs，此时不得不中转开胸，极少数病灶即使在开胸手术中也无法找到。

VATS术前CT引导下Hook-wire定位，简单易行，即使病灶很小，位置很深，这个方法也非常适合^[3-8]^，且定位成功率高，文献^[8-12]^报道在96%以上。本组患者定位成功率为100%，全组患者均先行楔形切除，根据快速冰冻病理结果决定是否行肺叶切除。其中4例患者定位后出现气胸，均为微量，未予处理，无出血病例，其中1例Hook-wire脱落。CT引导下的Hook-wire定位属于有创的定位方法，部分并发症不可避免，主要为气胸及定位针脱落移位。气胸的发生率为7.5%-49.1%^[13]^，气胸一般为微量，绝大多数不需要胸腔引流处理。Hook-wire脱落移位的发生率为4%-22%，仍可以根据穿刺形成的局部胸膜下血肿辨别病灶的准确位置，并成功进行切除，而不需要中转开胸^[9-12]^。Hook-wire的脱落移位与多种因素有关，包括所使用的Hook-wire的种类、所定位结节的数量、Hook-wire与病灶的位置关系以及病灶与壁层胸膜间的距离等^[11, 12]^。定位针位于病灶中心或穿透病灶者不易脱落移位。本组中有1例脱落，也是由于病灶紧贴脏层胸膜下。因此，病灶位置浅、定位针倒钩不能充分打开可能是发生脱落移位的主要原因。周建华等^[3]^单纯运用Hook-wire对100例110枚SPNs定位，成功率为100%，微量气胸19例，未予处理，VATS肺楔形切除98例，2例因为广泛粘连而中转开胸，6例发生Hook-wire脱落，但仍于胸腔镜下观察到穿刺点脏层胸膜下血肿后准确定位并成功切除。

CT引导下肺结节的Hook-wire定位的适应症：开始这项技术的初期，为了熟悉这项技术，我们选择了数枚实性结节，随着技术的熟练，我们严格了适应证的选择，主要以GGOs为主。在临床工作中我们体会到直径10 mm以上的实性结节或者实性成分的直径10 mm以上的mGGOs，都可以通过手指触诊发现。对于Hook-wire术前定位的适应症目前还没有统一标准，临床医师应根据结节的性质及距离胸膜的距离综合判断，我们认为以下患者使用该技术将会受益：①GGOs≤20 mm，实性结节≤10 mm；②结节位于肺野外1/3；③结节距脏层胸膜较远，如实性结节距脏层胸膜距离＞10 mm，GGOs距脏层胸膜距离＞5 mm等等；④结节不与脏层胸膜相连(无胸膜凹陷)。

通过CT引导下肺结节的Hook-wire定位及肺楔形切除术的临床应用，我们取得较好的临床效果，也总结了较多的临床经验：①定位前需充分与患者沟通并镇静止痛，防止定位过程中患者改变体位，从而导致定位失败。我们的做法是给所有患者定位前肌肉注射哌替啶50 mg。②定位前胸部CT扫描，根据病灶位置选择合适的体位、穿刺部位，确定进针的深度以及最佳的进针角度和路径，注意病灶位置不同，相应体位、穿刺部位、进针深度及角度均不同。③穿刺进针时，应避开胸廓骨性结构并尽可能遵循垂直最近原则，即定位针置入后与壁层胸膜的角度要尽量接近90度，单肺通气肺萎陷后定位针与胸壁间的摩擦力最小，不易造成Hook-wire脱落。④对于距脏层胸膜＜10 mm的表浅病灶，要求Hook-wire置入点必需超过病灶边缘5 mm以上，以防止Hook-wire脱落移位。⑤对于特殊部位的病灶，如紧贴主动脉弓等大血管的病灶，我们也是从侧胸壁穿刺置入Hook-wire，达到病灶但不穿过病灶，可以避免损伤血管，术中在监视下再将Hook-wire从肿瘤侧拉出并完成定位。⑥定位完成后应尽快手术，在翻身摆体位时应该注意保护Hook-wire，以免脱落移位。在楔形切除前应使用卵圆钳试夹，以确定Hook-wire不被夹入切割闭合器内，并保证距离肿瘤边缘2 cm以上为切除范围。Hook-wire定位后可以手指触到的结节，为方便切除，可以先拔出Hook-wire再进行切除。对于手指无法触到的结节，尽量避免先拔出Hook-wire再进行切除的做法，因为无法保证结节在楔形切除范围内，另外对于较小的GGOs即使在楔形切除范围内，也不一定保证仅频触觉和肉眼就能在术后标本中找到，所以保留Hook-wire可以起到指示的作用，方便术后病理医师取材。

随着高分辨CT的普及，肺部GGOs的检出率逐年增加。GGOs在CT上表现为局部肺的密度轻度增高，呈边界模糊或清楚的磨玻璃影，其内仍可以观察到血管及支气管影。根据其内是否含有实性成分将其分为pGGOs和mGGOs，pGGOs表现为均匀的磨玻璃密度病灶，而mGGOs表现为磨玻璃病灶内伴有条片状或带状高密度影。GGOs的病理基础为肺泡内气体减少，细胞数量增多，肺泡上皮细胞增生，肺泡间隔增厚和终末气囊内部分液体充填。其中，pGGOs是病变组织沿肺泡壁伏壁生长，不伴有肺泡结构的破坏，比如炎症、局灶性肺出血、AAH等；当病理组织增多，肺泡结构塌陷，成纤维细胞增生时，病灶逐步演变为含实性成分的mGGOs，如原位腺癌、浸润性腺癌、肺间质纤维化等^[14-15]^。对GGOs及病理之间的关系研究很多^[16-18]^，GGOs尤其是含有实性成分的mGGOs具有较高的恶性率，多数是原位腺癌，亦可是微浸润腺癌，甚至是浸润腺癌，但外科处理后预后良好。另有研究^[19]^显示持续存在的稳定的pGGOs恶性概率高达59%。Shimada等^[20]^的研究发现，如果GGOs病灶大小超过25 mm或者实性成分多以及影像学上提示侵袭性，那么该病灶是恶性肿瘤的几率升高且预后差。另国内文献报道^[21]^肺部＜10 mm的GGOs的恶性比例高达约68%。本组患者GGOs总的恶性比例是45%，mGGOs恶性比例是50%，与国外文献基本一致，pGGOs恶性比例是25%，由于本组pGGOs例数少，统计学差异不大。因此mGGOs或者有以下特点的pGGOs应予以积极的外科处理：影像学提示恶性程度高(如分叶、毛刺、血管集束征等)；或者观察期间结节增大或者实性成分增多；或者经抗炎治疗后无明显变化或持续存在；或者有肺癌危险因素，如高龄、家族史或者恶性肿瘤病史、吸烟史等。

综上所述，GGOs尤其是mGGOs恶性比例高，应给予积极的外科处理。SPNs尤其是GGOs术前运用Hook-wire定位的方法准确率高，可提高VATS肺楔形切除术的成功率；精确切除避免了多余肺组织的切除，最大限度保留了肺功能；并发症小，创伤小，促进了患者术后的快速康复，值得推广。
